# The safety and effectiveness of pegfilgrastim to reduce cancer chemotherapy-induced febrile neutropenia in real-world practice in Japan: a post-marketing surveillance study

**DOI:** 10.1007/s00520-025-10042-6

**Published:** 2025-11-22

**Authors:** Nobuhiro Shibata, Hiroshi Kuwazawa, Tomoharu Yasukawa, Manabu Iwabuchi, Shigehira Saji

**Affiliations:** 1 Department of Clinical Oncology, Kansai Medical University Hospital, Hirakata, Japan; 2 Cancer Treatment Center, Kansai Medical University Hospital, Hirakata, Japan; 3 Kyowa Kirin Co., Ltd, Tokyo, Japan; 4 Department of Medical Oncology, Fukushima Medical University, Fukushima, Japan

**Keywords:** Febrile neutropenia, Chemotherapy, Pegfilgrastim, G-CSF, Post-marketing surveillance, Real-world data

## Abstract

**Purpose:**

We performed post-marketing surveillance of the safety and effectiveness of 3.6 mg pegfilgrastim to prevent chemotherapy-induced febrile neutropenia (FN) in real-world conditions in Japan.

**Methods:**

Patients were registered between June 2015 and May 2017 and followed prospectively. Pegfilgrastim was administered once every chemotherapy cycle (maximum 6 cycles). Use of pegfilgrastim, safety, and effectiveness in reducing FN were evaluated.

**Results:**

From 300 institutions, 1,597 patients were registered and 1,479 patients were analyzed. Pegfilgrastim was given as primary prophylaxis (750 patients), as secondary prophylaxis (727 patients), or for therapeutic purposes (2 patients). The most common primary diseases were breast cancer (51.4%) and non-Hodgkin lymphoma (25.6%). Adverse events (AEs) occurred in 36.4% of patients and adverse drug reactions (ADR) in 18.5%. Common ADRs included back pain (3.6%), pyrexia (3.1%), arthralgia (2.1%), hepatic function abnormal (1.5%), myalgia (1.4%), and bone pain (1.0%). All back and/or bone pain-related ADRs were non-serious and well-controlled with non-steroidal anti-inflammatory drugs. Docetaxel-cyclophosphamide therapy among breast cancer patients was a factor influencing bone and/or back pain-related AEs by multivariate logistic regression analysis (odds ratios vs. fluorouracil-epirubicin-cyclophosphamide therapy: 2.32, P = 0.031).

FN was observed in 5.3% (primary prophylaxis) and 2.5% (secondary prophylaxis) of the effectiveness analysis set in cycle 1 and decreased with increasing cycles.

**Conclusion:**

This survey confirmed that both primary and secondary prophylaxis using pegfilgrastim reduced FN in the real-world setting. No new safety concerns were identified.

This survey was retrospectively registered on 26 April 2024 in the University Hospital Medical Information Network Clinical Trial Registry (UMIN000054267).

**Supplementary Information:**

The online version contains supplementary material available at 10.1007/s00520-025-10042-6.

## Introduction

Febrile neutropenia (FN) often develops after myelosuppressive cancer chemotherapy, resulting in hospitalization or extension of the hospital stay. Thus, FN is one of the dose-limiting toxicities that often lead to dose reduction or discontinuation of the cancer therapy [1]. Managing FN is critical to maximize the effectiveness of chemotherapy.

Pegfilgrastim is a pegylated filgrastim that was developed by covalently conjugating the 20 kDa monomethoxypolyethylene glycol molecule to recombinant human granulocyte colony-stimulating factor (G-CSF, or filgrastim) [2, 3]. The serum half-life of filgrastim is extended by pegylation; thus, pegfilgrastim administration is required only once per cycle in patients undergoing chemotherapy. Pegfilgrastim effectively reduces the risk of FN in cancer patients under chemotherapy. After approval of pegfilgrastim in other countries in 2002, clinical trials started in Japan. In phase I studies, the pharmacokinetics and pharmacodynamics of pegfilgrastim were confirmed to be similar to those in previous clinical studies conducted in other countries [4]. In phase II studies, pegfilgrastim was evaluated in patients with non-Hodgkin lymphoma (NHL) or Hodgkin lymphoma (HL) undergoing cyclophosphamide-cytarabine-etoposide-dexamethasone ± rituximab (CHASE(R)) therapy, as well as patients with breast cancer undergoing docetaxel-doxorubicin-cyclophosphamide (TAC) therapy [4, 5]. From these studies, the optimal dose of pegfilgrastim for Japanese patients was determined to be a single injection of 3.6 mg per cycle. This dose is lower than that commonly used in other countries (6 mg) [6, 7]. In a placebo-controlled, double-blind, randomized trial in Japanese breast cancer patients receiving docetaxel-cyclophosphamide (TC) therapy, significant suppression of FN by pegfilgrastim was confirmed (pegfilgrastim group 1.2% (2/173 cases), placebo group 68.8% (119/173 cases), P < 0.001) [8]. Another randomized, double-blind phase III study in patients with NHL or HL undergoing CHASE(R) therapy used the duration of severe neutropenia (days in which absolute neutrophil count was < 500/μL in the first cycle) as the primary outcome and demonstrated non-inferiority to filgrastim (pegfilgrastim 4.5 ± 1.2 days vs. filgrastim 4.7 ± 1.3 days, mean ± standard deviation (SD)) [9]. Furthermore, a randomized, open-label phase III study in elderly patients (aged > 65 years) with NHL undergoing cyclophosphamide-doxorubicin-vincristine-prednisone ± rituximab CHOP(R) therapy showed the FN (defined as fever ≥ 37.5 and absolute neutrophil count ≤ 500/μL) was 1/25 patients (4.0%) with pegfilgrastim vs. 5/27 patients (18.5%) with filgrastim [4].These clinical studies demonstrated the safety and efficacy of pegfilgrastim and led to its approval in 2014 in Japan for the suppression of FN in patients undergoing cancer chemotherapy.

The safety and efficacy of pegfilgrastim have been tested mostly in patients with breast cancer or NHL. Notably, pegfilgrastim is approved for patients with any type of cancer because its efficacy has been demonstrated in malignant lymphoma as well as breast cancer [8]. However, robust clinical trials are lacking that support the efficacy of pegfilgrastim in various cancer types other than breast cancer and NHL. Nonetheless, G-CSF shows efficacy in reducing the FN risk among patients with various solid tumors and lymphoma, other than breast cancer or NHL, undergoing chemotherapy [10]. Furthermore, primary outcomes of clinical studies in Japan and abroad include duration of severe neutropenia, except in one Japanese clinical study where FN frequency was the primary outcome [8]. Therefore, the purpose of this post-marketing surveillance study was to describe the safety and efficacy of pegfilgrastim to suppress FN following cancer chemotherapy in the real-world setting, where pegfilgrastim is used to treat patients with various types of cancer and chemotherapy regimens, and whose safety and efficacy data might be insufficiently collected in clinical studies. We also evaluated the current use of pegfilgrastim and its efficacy as a primary and secondary prophylaxes.

## Methods

### Patients and study design

This study was conducted as a post-marketing surveillance study to confirm the safety and effectiveness of pegfilgrastim (G-Lasta®, Kyowa Kirin Co., Ltd, Tokyo, Japan) in all types of cancers.

The targeted patients were Japanese cancer patients who received a first administration of pegfilgrastim as supportive care to reduce chemotherapy-induced FN. Registration was conducted from June 2015 to May 2017, with a plan to include 1,500 patients. During the period after pegfilgrastim administration was scheduled and before the first administration, patients were registered centrally and followed prospectively. The recommended dosage of pegfilgrastim was a single subcutaneous injection of 3.6 mg at > 24 h after chemotherapy completion, with one administration per chemotherapy cycle in a maximum of 6 cycles. The dose of 3.6 mg, instead of 6.0 mg as commonly used worldwide, is recommended in Japan following the results of a dose–response study conducted in Japanese NHL or HL [4] and breast cancer patients [5]. After the last or 6th pegfilgrastim administration, whichever was earlier, the follow-up observation was completed at the beginning of the next chemotherapy cycle. If there was no following chemotherapy, follow-up observation was completed 1 month after the last administration of pegfilgrastim.

Patients received pegfilgrastim as the primary or secondary prophylaxis. The two prophylactic administrations are defined with the timing of administration in the G-CSF clinical guidelines by the Japanese Society of Medical Oncology [11]. However, the timing of use is interpreted differently among physicians in real practice. Therefore, in this survey, pegfilgrastim administration in the first cycle of chemotherapy was defined as the primary prophylaxis and administration in the second or later cycles was defined as the secondary prophylaxis.

### Safety

Among the initially registered patients, those who met one of the following criteria were excluded: the first administration of pegfilgrastim was before the date of registration; the first administration was before the date that the institution signed the survey agreement; repeated registration of one patient by different physicians; pegfilgrastim administration did not take place; history of previous administration of pegfilgrastim; and safety could not be evaluated. The remaining patients were subjected to safety evaluation (the safety analysis set).

Adverse events (AEs) were recorded using System Organ Classes and Preferred Terms of MedDRA/J version 22.1. The number of patients who experienced each AE at least once was counted. Seriousness and the causal relationship with pegfilgrastim were evaluated by reporting physicians. The event was recorded as an adverse drug reaction (ADR), unless causality owing to pegfilgrastim was ruled out. AEs of special interest were as follows: interstitial lung disease, splenomegaly/splenic rupture, shock/anaphylaxis, acute respiratory distress syndrome, blasts increased, capillary leak syndrome, bone and/or back pain-related AEs, Sweet’s syndrome, cutaneous vasculitis, and large vessel vasculitis. Serious thrombocytopenia and secondary malignancy were defined as a potential AE of special interest.

### Effectiveness

From the safety analysis set, patients with the following conditions were excluded: efficacy evaluation was not possible; and pegfilgrastim was used for other than chemotherapy-induced FN or without chemotherapy (off-label use). The remaining patients were subjected to effectiveness analysis (the efficacy analysis set). The primary outcome was FN frequency. The factors influencing chemotherapy-associated FN were analyzed.

### Statistical analysis

Patient characteristics and safety information were described in summary statistics. Factors that may influence the occurrence of FN and bone and/or back pain-related AEs were evaluated using logistic regression analyses. Candidate variables were selected from those identified as clinically important in published guidelines and the literature. Univariate or multivariate logistic regression analyses were conducted using Firth’s penalized likelihood approach; multivariate models were constructed using the forced-entry method. Odds ratios (ORs) with 95% confidence intervals (CI) were determined. 95% CI and P-values were calculated using the Wald method. In post-hoc subgroup analysis, patients were classified by cancer type and chemotherapy regimens, and FN incidence rates and their 95% CIs were calculated.

The statistical analysis plan was completed before locking the data. All statistical analyses were performed independently by CMIC Co., Ltd (Tokyo, Japan) based on the statistical analysis plan, except a post-hoc analysis performed after the planned analyses were completed.

## Results

### Patient characteristics

During the survey period, 1,597 patients were registered from 300 institutions. Among these patients, 29 patients were excluded because the survey forms were not submitted, 53 patients from 10 institutions were excluded because of lack of consent for the publication of survey results from the institution, and 36 patients were excluded because of the safety analysis exclusion criteria described in the Methods. The remaining 1,479 patients were included in the safety analysis set (Fig. S1). From these patients, 8 patients with off-label use or missing key data were excluded, leaving 1,471 patients in the effectiveness analysis set.

The patient characteristics of the safety analysis set (N = 1,479) are summarized in Table 1. There were 450 male patients (30.4%), and the median age (min, max) at the start of administration was 64.0 (14, 95) years; approximately 46.9% (n = 693) were ≥ 65 years old. The number of patients who received pegfilgrastim as primary prophylaxis was 750 and the number of patients who received pegfilgrastim as secondary prophylaxis was 727. The main primary diseases were breast cancer (760 patients, 51.4%), NHL (379 patients, 25.6%), non-small cell lung cancer (NSCLC) (87 patients, 5.9%), and small cell lung cancer (SCLC) (77 patients, 5.2%). Otherwise, patients with 29 other types of cancer were treated with pegfilgrastim; cancer types in ≥ 10 patients were prostate cancer, soft tissue sarcoma, esophageal carcinoma, head and neck cancer, colorectal cancer, and uterine cancer. Most patients had a performance status (PS) of 0 (1,016 patients, 68.7%) and 96 patients (6.5%) had PS 2 or higher.

**Table 1:** Patient characteristics: Safety analysis set (N = 1479)

	All patients^a^ N (%)	Primary prophylaxis N (%)	Secondary prophylaxis N (%)
Safety analysis set	1479 (100)	750 (100)	727 (100)
Sex			
Male	450 (30.4)	182 (24.3)	266 (36.6)
Female	1029 (69.6)	568 (75.7)	461 (63.4)
Age [years]			
Median (min, max)	64.0 (14, 95)	62.0 (14, 89)	65.0 (19, 95)
< 65	786 (53.1)	423 (56.4)	362 (49.8)
≥ 65	693 (46.9)	327 (43.6)	365 (50.2)
Primary disease			
Breast cancer	760 (51.4)	498 (66.4)	262 (36.0)
NHL	379 (25.6)	111 (14.8)	266 (36.6)
NSCLC	87 (5.9)	36 (4.8)	51 (7.0)
SCLC	77 (5.2)	19 (2.5)	58 (8.0)
Others	176 (11.9)	86 (11.5)	90 (12.4)
Performance status			
0	1016 (68.7)	571 (76.1)	444 (61.1)
1	360 (24.3)	136 (18.1)	224 (30.8)
≥ 2	96 (6.5)	38 (5.1)	57 (7.8)
unknown	7 (0.5)	5 (0.7)	2 (0.3)
Comorbidity			
Absent	751 (50.8)	431 (57.5)	320 (44.0)
Present ^b^	728 (49.2)	319 (42.5)	407 (56.0)
Infectious disease	67 (4.5)	24 (3.2)	43 (5.9)
Cardiac disorder	79 (5.3)	26 (3.5)	53 (7.3)
Lung disorder	63 (4.3)	26 (3.5)	37 (5.1)
Hepatic disorder	55 (3.7)	27 (3.6)	28 (3.9)
Renal disorder	12 (0.8)	5 (0.7)	7 (1.0)
Previous treatment for the primary disease^a^			
None	391 (26.4)	389 (51.9)	0 (0.0)
Performed	1088 (73.6)	361 (48.1)	727 (100.0)
Chemotherapy within the last 3 months			
None	622 (42.1)	620 (82.7)	0 (0.0)
Performed	857 (57.9)	130 (17.3)	727 (100.0)
The same regimen for which pegfilgrastim was administered			
	727 (49.2)	0 (0.0)	727 (100.0)
Radiotherapy			
None	1395 (94.3)	710 (94.7)	683 (93.9)
Performed	84 (5.7)	40 (5.3)	44 (6.1)
Surgery			
None	1159 (78.4)	523 (69.7)	634 (87.2)
Performed	320 (21.6)	227 (30.3)	93 (12.8)

^a^ Safety analysis set includes 2 patients whose pegfilgrastim use was not prophylactic but therapeutic against febrile neutropenia

^b^ Multiple counts on a single patient possible

*NHL* non-Hodgkin lymphoma, *NSCLC* non-small cell lung cancer, *SCLC* small cell lung cancer

A large proportion of patients (1,088 patients, 73.6%) underwent previous treatment for the primary disease, including surgery in 320 patients (21.6%), radiation therapy in 84 patients (5.7%), and cancer chemotherapy within the last 3 months in 857 patients (57.9%). At the time of pegfilgrastim initiation, 727 patients (49.2%) were under chemotherapy and they continued the same regimen during pegfilgrastim administration.

Cancer chemotherapy regimens implemented in the first cycle are summarized in Table 2. In the 760 breast cancer patients, the common regimens were doxorubicin-cyclophosphamide/epirubicin-cyclophosphamide (AC/EC) therapy (216/760 patients, 28.4%), fluorouracil-epirubicin-cyclophosphamide (FEC) therapy (215/760 patients, 28.3%), and TC therapy (208/760 patients, 27.4%). For the 379 NHL patients, R-CHOP therapy (148/379 patients, 39.1%) and cyclophosphamide-doxorubicin-vincristine-prednisone (CHOP) therapy (44/379 patients, 11.6%) were commonly used. For NSCLC treatment (87 patients), docetaxel monotherapy was used in 19/87 patients (21.8%), docetaxel/ramucirumab was used in 11/87 patients (12.6%), and carboplatin/pemetrexed/bevacizumab was used in 10/87 patients (11.5%). For SCLC patients (77 patients), carboplatin/etoposide therapy (30/77 patients, 39.0%), amrubicin monotherapy (22/77 patients, 28.6%), and cisplatin/etoposide therapy (15/77 patients, 19.5%) were commonly used.The administration of pegfilgrastim was conducted almost exactly as recommended (3.6 mg subcutaneous injection on the day after chemotherapy completion or later). In the first cycle, all 1,479 patients received 3.6 mg. The median period from the completion of chemotherapy until pegfilgrastim administration was 3 days for any cycle, with most administrations made between the 2nd and 4th days after chemotherapy completion (87.3% to 90.5%). In each cycle, 0%–2.25% of patients received pegfilgrastim on the same day as chemotherapy completion. Furthermore, nine patients received pegfilgrastim during their chemotherapy cycle. There was apparently no bias in cancer types or chemotherapy regimens among these patients.

**Table 2:** Chemotherapy performed at the first cycle: Safety analysis set (N = 1479)

Chemotherapy	All patients^a^ N (%)	Primary prophylaxis N (%)	Secondary prophylaxis N (%)
Safety analysis set	1479 (100)	750 (100)	727 (100)
Breast cancer	760 (51.4)	498 (66.4)	262 (36.0)
FEC therapy	215 (28.3)	127 (25.5)	88 (33.6)
TC therapy	208 (27.4)	167 (33.5)	41 (15.6)
AC/EC therapy	216 (28.4)	137 (27.5)	79 (30.2)
Q2W	44 (5.8)	42 (8.4)	2 (0.8)
Q3W	138 (18.2)	81 (16.3)	57 (21.8)
≥ Q4W	17 (2.2)	7 (1.4)	10 (3.8)
Unknown	17 (2.2)	7 (1.4)	10 (3.8)
NHL	379 (25.6)	111 (14.8)	266 (36.6)
R-CHOP therapy	148 (39.1)	40 (36.0)	108 (40.6)
CHOP therapy	44 (11.6)	17 (15.3)	27 (10.2)
NSCLC	87 (5.9)	36 (4.8)	51 (7.0)
DTX monotherapy	19 (21.8)	5 (13.9)	14 (27.5)
DTX/RAM	11 (12.6)	6 (16.7)	5 (9.8)
CBDCA/PEM/BV	10 (11.5)	5 (13.9)	5 (9.8)
SCLC	77 (5.2)	19 (2.5)	58 (8.0)
CBDCA/VP-16	30 (39.0)	8 (42.1)	22 (37.9)
AMR monotherapy	22 (28.6)	8 (42.1)	14 (24.1)
CDDP/VP-16	15 (19.5)	3 (15.8)	12 (20.7)

^a^ Safety analysis set includes 2 patients whose pegfilgrastim use was not prophylactic but therapeutic against febrile neutropenia

Proportions of primary disease were calculated as percentages in the safety analysis set. Proportions of chemotherapy were calculated as percentages in patients with the primary disease

*AC* doxorubicin hydrochloride and cyclophosphamide hydrate; *AMR* amrubicin hydrochloride; *BV* bevacizumab; *CBDCA* carboplatin; *CDDP* cisplatin; *CHOP* cyclophosphamide hydrate, doxorubicin hydrochloride, vincristine sulfate, and prednisone; *DTX* docetaxel hydrate; *EC* epirubicin hydrochloride; *FEC* fluorouracil, epirubicin hydrochloride, and cyclophosphamide hydrate; *NHL* non-Hodgkin lymphoma; *NSCLC* non-small cell lung cancer; *PEM* pemetrexed sodium hemipentahydrate; *Q2W* every 2 weeks; *Q3W* every 3 weeks; *RAM* ramucirumab; *R-CHOP* rituximab, cyclophosphamide hydrate, doxorubicin hydrochloride, vincristine sulfate, and prednisone; *SCLC* small cell lung cancer; *TC* docetaxe hydrate and cyclophosphamide hydrate; *VP-16* etoposide

### Safety

The safety information of patients in the safety analysis set (N = 1,479) is summarized in Table S1. A total of 1,294 AEs occurred in 538 patients (36.4%), 185 serious AEs occurred in 116 patients (7.8%), and 7 AEs leading to death occurred in 6 patients (0.4%). Furthermore, 530 ADRs occurred in 274 patients (18.5%), 34 serious ADRs occurred in 28 patients (1.9%), and 1 ADR leading to death occurred in 1 patient (0.1%).

AEs observed in more than 2.0% of all patients included FN (83 patients 5.6%), pyrexia (64 patients, 4.3%), back pain (57 patients, 3.9%), nausea (46 patients, 3.1%), constipation (43 patients, 2.9%), anemia, arthralgia (39 patients, 2.6%, each), platelet count decreased (35 patients, 2.4%), neutrophil count decreased (31 patients, 2.1%), stomatitis, and hepatic function abnormal (29 patients, 2.0%, each). Serious AEs observed in ≥ 5 patients included FN (42 patients, 2.8%), white blood cell count decreased (8 patients, 0.5%), pyrexia, platelet count decreased, interstitial lung disease (6 patients, 0.4% each), herpes zoster, neutrophil count decreased, pneumonia, and decreased appetite (5 patients, 0.3% each).

ADRs observed in more than 1.0% of all patients included back pain (53 patients, 3.6%), pyrexia (46 patients, 3.1%), arthralgia (31 patients, 2.1%), hepatic function abnormal (22 patients, 1.5%), myalgia (20 patients, 1.4%), and bone pain (15 patients, 1.0%). Serious ADRs observed in ≥ 2 patients included pyrexia and interstitial lung disease (5 patients, 0.3% each), FN (4 patients, 0.3%), urticaria and platelet count decreased (2 patients, 0.1% each).

AEs that resulted in death were hepatic function abnormal, FN, cerebral infarction, shock hemorrhagic, respiratory arrest, and pneumonia bacterial/pneumocystis jirovecii pneumonia in 6 patients. All AEs leading to death were considered unrelated to pegfilgrastim, except one case with respiratory arrest recorded as an ADR. The patient had early-stage breast cancer (PS 0, stage III). The patient received pegfilgrastim on the second day of the third cycle of FEC therapy and died 3 days after pegfilgrastim administration. This patient had hypertension as a comorbidity and received a hypotensive agent before FEC therapy initiation. The exact cause of respiratory arrest remained undetermined; cerebrovascular or cardiovascular events could not be ruled out because of the comorbidity. Therefore, the event was reported as a serious ADR because of an unknown causal relationship with pegfilgrastim.

### AEs of special interest

Bone and/or back pain-related AEs were observed together in 67/1,479 patients (4.5%), including 40/750 patients (5.3%) in the primary prophylactic group and 27/727 patients (3.7%) in the secondary prophylactic group (Table 3). All events were classified as non-serious and a causal relationship with pegfilgrastim could not be ruled out in 63/67 patients (94%). Bone and/or back pain-related AEs were treated in 37/67 patients (55.2%), most commonly with loxoprofen (19/67 patients, 28.4%), acetaminophen (16/67 patients, 23.9%), and non-steroidal anti-inflammatory drugs (NSAIDs) other than loxoprofen (7/67 patients, 10.4%). The onset of bone and/or back pain-related AEs was high in cycle 1 (52/67 patients, 77.6%). The time from pegfilgrastim administration to the onset was 4.4 ± 5.1 days (mean ± SD).

**Table 3:** Adverse events of special interest (bone and/or back pain-related adverse events): Safety analysis set (N = 1479)

	All patients^a^ N (%)	Primary prophylaxis N (%)	Secondary prophylaxis N (%)
Safety analysis set	1479 (100)	750 (100)	727 (100)
Bone and/or back pain-related adverse events			
	67 (4.5)	40 (5.3)	27 (3.7)
Seriousness (evaluated by physician)			
Serious	0 (0.0)	0 (0.0)	0 (0.0)
Non-serious	67 (100.0)	40 (100.0)	27 (100.0)
Causal relationship (evaluated by physician)			
No	4 (6.0)	2 (5.0)	2 (7.4)
Yes	56 (83.6)	36 (90.0)	20 (74.0)
Unknown	7 (10.4)	2 (5.0)	5 (18.5)
Related factors other than pegfilgrastim			
No	35 (52.2)	18 (45.0)	17 (63.0)
Yes	32 (47.8)	22 (55.0)	10 (37.0)
Treatment for bone and/or back pain-related adverse events			
No	30 (44.8)	16 (40.0)	14 (51.9)
Yes	37 (55.2)	24 (60.0)	13 (48.1)
Loxoprofen	19 (28.4)	13 (32.5)	6 (22.2)
Acetaminophen	16 (23.9)	10 (25.0)	6 (22.2)
NSAIDs^b^	7 (10.4)	4 (10.0)	3 (11.1)
Chinese medicine	1 (1.5)	1 (2.5)	0 (0.0)
Treatment cycle at the onset of bone and/or back pain-related adverse events			
1	52 (77.6)	33 (82.5)	19 (70.4)
2	7 (10.4)	2 (5.0)	5 (18.5)
≥ 3	8 (11.9)	5 (12.5)	3 (11.1)
Days until onset of bone and/or back pain-related AEs^c^			
Mean ± SD	4.4 ± 5.1	3.5 ± 2.1	5.8 ± 7.5
Median (min, max)	4.0 (−2, 33)	3.0 (0, 8)	4.0 (−2, 33)
1	10 (14.9)	5 (12.5)	5 (18.5)
2–7	44 (65.7)	32 (80.0)	12 (44.4)
8–14	6 (9.0)	2 (5.0)	4 (14.8)
≥ 15	2 (3.0)	0 (0.0)	2 (7.4)

^a^ Safety analysis set includes 2 patients whose pegfilgrastim use was not prophylactic but therapeutic against febrile neutropenia

^b^ Except loxoprofen

^c^ Days were counted from the start of pegfilgrastim treatment of the cycle, in which the bone and/or back pain-related AEs occurred

*AEs* adverse events, *NSAIDs* non-steroidal anti-inflammatory drugs, *SD* standard deviation

Multivariate logistic regression analysis was performed for bone and/or back pain-related AEs (Table 4). Multivariate logistic regression analysis revealed that the chemotherapy regimen TC therapy for breast cancer patients significantly influenced the onset of bone and/or back pain-related AEs (OR vs. FEC therapy as reference: 2.32, P = 0.031).

**Table 4:** Multivariate logistic regression analysis for bone and/or back pain-related adverse events: Safety analysis set (N = 1479)

	Patients N	AE occurrence N (%)	Univariate analysis			Multivariate analysis		
			Odds ratio	95% CI	P-value	Odds ratio	95% CI	P-value
Patients analyzed^a^	1470	67 (4.6)						
Sex								
Male	445	13 (2.9)	Reference	−	−	Reference	−	−
Female	1025	54 (5.3)	1.80	0.98–3.30	0.058	0.77	0.35–1.66	0.497
Age (years)								
< 65	784	47 (6.0)	Reference	−	−	Reference	−	−
≥ 65	686	20 (2.9)	0.48	0.28–0.81	0.006^**^	0.59	0.34–1.02	0.061
Chemotherapy regimen								
Breast cancer								
FEC	214	10 (4.7)	Reference	−	−	Reference	−	−
TC	208	22 (10.6)	2.35	1.10–5.03	0.028*	2.32	1.08–4.96	0.031*
AC/EC	216	10 (4.6)	0.99	0.41–2.39	0.983	0.96	0.40–2.29	0.922
NHL								
R-CHOP/CHOP	192	4 (2.1)	0.46	0.15–1.43	0.182	0.54	0.16–1.81	0.314
NSCLC/SCLC								
Platinum-based	93	1 (1.1)	0.32	0.06–1.79	0.193	0.29	0.05–1.79	0.184
Non-platinum-based	71	0 (0.0)	0.14	< 0.01–2.40	0.173	0.13	< 0.01–2.29	0.165
Other	476	20 (4.2)	0.87	0.41–1.88	0.731	0.93	0.40–2.19	0.876
Performance status								
0	1015	55 (5.4)	Reference	−	−	Reference	−	−
1	360	11 (3.1)	0.57	0.30–1.09	0.088	0.79	0.40–1.57	0.495
≥ 2	95	1 (1.1)	0.27	0.05–1.42	0.123	0.37	0.07–1.86	0.228
Comorbidity (number of organ disorders)								
0	1288	58 (4.5)	Reference	−	−	Reference	−	−
1	139	7 (5.0)	1.19	0.54–2.61	0.663	1.87	0.85–4.15	0.122
≥ 2	43	2 (4.7)	1.27	0.34–4.73	0.725	3.35	0.88–12.74	0.076
Reason for prescription								
Primary prophylaxis	745	40 (5.4)	Reference	−	−	Reference	−	−
Secondary prophylaxis	725	27 (3.7)	0.69	0.42–1.13	0.136	0.98	0.59–1.64	0.941

^a^ From the safety analysis set (N = 1479), nine patients with missing dependent variables were excluded

^*^ P < 0.05; ** P < 0.01

*AC* doxorubicin hydrochloride and cyclophosphamide hydrate; *AE* adverse event; *CHOP* cyclophosphamide hydrate, doxorubicin hydrochloride, vincristine sulfate, and prednisone; *CI* confidence interval; *EC* epirubicin hydroclolide and cyclophosphamide hydrate; *FEC* fluorouracil, epirubicin hydroclolide, and cyclophosphamide hydrate; *NHL* non-Hodgkin lymphoma; *NSCLC* non-small cell lung cancer; *R-CHOP* rituximab, cyclophosphamide hydrate, doxorubicin hydrochloride, vincristine sulfate, and prednisone; *SCLC* small cell lung cancer; *TC* docetaxel hydrate and cyclophosphamide hydrate

Thrombocytopenia information by chemotherapy regimen is summarized in Table S2. Thrombocytopenia was observed in 43/1,479 patients (2.9%), including 17/750 patients (2.3%) in the primary prophylaxis group and 26/727 patients (3.6%) in the secondary prophylaxis group. The mean lowest platelet count ± SD was 6.50 ± 3.84 × 10^4^/µL, and the platelet count dropped to Grade 3 in 11/43 patients (25.6%) and Grade 4 in 7/43 patients (16.3%). The lowest platelet count was 9.0 ± 11.2 days (mean ± SD) after the administration of pegfilgrastim. There was a higher frequency of thrombocytopenia in patients who underwent platinum-based chemotherapy regimens than those who underwent regimens without platinum-based chemotherapy in the total and secondary prophylaxis populations.

Other ADRs of special interest, such as interstitial lung disease (6 patients), blasts increased (4 patients), secondary malignancy (1 patient), and Sweet's syndrome (1 patient) were also observed. For these ADRs, a causal relationship was suspected with chemotherapies and a definite causal relationship with pegfilgrastim was considered unlikely. No patients experienced splenomegaly/splenic rupture, shock/anaphylaxis, acute respiratory distress syndrome, capillary leak syndrome, cutaneous vasculitis, or large vessel vasculitis.

### Effectiveness

The frequency of FN in primary and secondary prophylaxis groups is summarized in Table 5. In the primary prophylaxis group, the FN frequency in cycle 1 was 5.3% in all cancer patients, 16.0% in NHL patients, 7.3% in NSCLC/SCLC patients, and 2.8% in breast cancer patients. In further cycles, the FN frequency was low (0.7% to 1.7%) in all cancer patients. The trend was similar across cancer types. In the secondary prophylaxis group, the FN frequency in cycle 1 was 2.5% in all cancer patients, 3.3% in NHL patients, 2.4% in breast cancer patients, and 1.0% in NSCLC/SCLC patients. Consistent with results observed for primary prophylaxis, the FN frequency in further cycles was low, ranging from 0.0% to 2.6%, in all patient groups, and the trend was similar across cancer types. FN frequencies by chemotherapy regimen, for the entire period and first cycle, are summarized in Table S3. A univariate or multivariate logistic regression analysis was performed on FN frequency (Table 6). Factors significantly affecting FN frequency included hospital status (outpatient, 29/913 patients (3.2%) vs. inpatient, 50/551 patients (9.1%): OR 0.40, P < 0.001), PS (≥ 2, 14/94 patients (14.9%) vs. 0, 39/1,012 patients (3.9%): OR 3.14, P = 0.003), history of FN (yes, 30/337 patients (8.9%) vs. no, 49/1,127 (4.3%): OR 3.21, P < 0.001), and reason for prescription (secondary prophylaxis, 34/721 (4.7%) vs primary prophylaxis, 45/743 patients (6.1%): OR 0.52, P = 0.024). The mean neutrophil counts ± SD before the initiation of chemotherapy were 3,686.6 ± 2,162.2/µL in the primary prophylaxis group and 3,325.9 ± 2,159.5/µL in the secondary prophylaxis group. After the initiation of pegfilgrastim, the neutrophil counts recovered and this recovery was observed at most cycles at and after cycle 2. The mean (± SD) neutrophil counts at the final observation were 4,716.5 ± 3,009.2/µL in the primary prophylaxis group and 4,236.2 ± 3,662.7/µL in the secondary prophylaxis group (Fig. S2). The same trend was demonstrated in patients with different cancer types and chemotherapy regimens in subgroup analyses; neutrophil counts recovered with pegfilgrastim in the second cycle and were maintained thereafter (Tables 5, S3).

**Table 5:** Frequency of febrile neutropenia: Effectiveness analysis set (N = 1471)

Febrile neutropenia frequency: prevalence/analyzed (%)								
	Patients N	Entire period	Cycle 1^a^	Cycle 2	Cycle 3	Cycle 4	Cycle 5	Cycle 6
Primary prophylaxis								
All cancer patients analyzed^b^	682	43/682 (6.3)	36/682 (5.3)	5/573 (0.9)	6/509 (1.2)	3/436 (0.7)	1/77 (1.3)	1/59 (1.7)
Breast cancer	472	16/472 (3.4)	13/472 (2.8)	2/438 (0.5)	3/408 (0.7)	1/357 (0.3)	0/18 (0.0)	0/14 (0.0)
FEC therapy	119	4/119 (3.4)	3/119 (2.5)	0/112 (0.0)	0/106 (0.0)	1/92 (1.1)	0/6 (0.0)	0/5 (0.0)
TC therapy	159	5/159 (3.1)	5/159 (3.1)	1/149 (0.7)	1/142 (0.7)	0/126 (0.0)	0/1 (0.0)	0/0 (0.0)
AC/EC therapy	132	5/132 (3.8)	3/132 (2.3)	0/125 (0.0)	2/115 (1.7)	0/101 (0.0)	0/1 (0.0)	0/1 (0.0)
NHL	94	17/94 (18.1)	15/94 (16.0)	2/58 (3.4)	3/46 (6.5)	1/37 (2.7)	1/32 (3.1)	1/25 (4.0)
NSCLC/SCLC	41	3/41 (7.3)	3/41 (7.3)	0/27 (0.0)	0/19 (0.0)	0/14 (0.0)	0/7 (0.0)	0/5 (0.0)
Secondary prophylaxis								
All cancer patients analyzed^b^	628	27/628 (4.3)	16/628 (2.5)	8/449 (1.8)	4/345 (1.2)	4/154 (2.6)	1/107 (0.9)	0/31 (0.0)
Breast cancer	249	11/249 (4.4)	6/249 (2.4)	4/188 (2.1)	2/138 (1.4)	0/10 (0.0)	0/5 (0.0)	0/3 (0.0)
FEC therapy	82	4/82 (4.9)	3/82 (3.7)	1/57 (1.8)	0/42 (0.0)	0/1 (0.0)	0/1 (0.0)	0/0 (0.0)
TC therapy	39	1/39 (2.6)	1/39 (2.6)	0/32 (0.0)	0/25 (0.0)	0/1 (0.0)	0/0 (0.0)	0/0 (0.0)
AC/EC therapy	76	5/76 (6.6)	1/76 (1.3)	3/65 (4.6)	2/46 (4.3)	0/4 (0.0)	0/1 (0.0)	0/1 (0.0)
NHL	212	13/212 (6.1)	7/212 (3.3)	4/156 (2.6)	2/130 (1.5)	4/104 (3.8)	1/74 (1.4)	0/15 (0.0)
NSCLC/SCLC	96	1/96 (1.0)	1/96 (1.0)	0/63 (0.0)	0/45 (0.0)	0/21 (0.0)	0/15 (0.0)	0/6 (0.0)

^a^ Cycle 1 was defined as the first cycle in which pegfilgrastim was initiated

^b^ From the effectiveness analysis set (N = 1471), patients who received pegfilgrastim within 5 days after the completion of each chemotherapy cycle were analyzed in this summary

*AC* doxorubicin hydrochloride and cyclophosphamide hydrate; *EC* epirubicin hydrochloride; *FEC* fluorouracil, epirubicin hydrochloride, and cyclophosphamide hydrate; *NHL* non-Hodgkin lymphoma; *NSCLC* non-small cell lung cancer; *SCLC* small cell lung cancer; *TC* docetaxel hydrate and cyclophosphamide hydrate

**Table 6:** Multivariate logistic regression analysis of febrile neutropenia frequency (adverse event): Effectiveness analysis set (N = 1471)

	Patients N	FN prevalence N (%)	Univariate analysis			Multivariate analysis		
			Odds ratio	95% CI	P-value	Odds ratio	95% CI	P-value
Patients analyzed^a^	1464	79 (5.4)						
Sex								
Male	442	33 (7.5)	Reference	−	−	Reference	−	−
Female	1022	46 (4.5)	0.58	0.37–0.92	0.021*	0.69	0.38–1.27	0.229
Age (years)								
< 65	781	36 (4.6)	Reference	−	−	Reference	−	−
≥ 65	683	43 (6.3)	1.39	0.88–2.18	0.157	1.05	0.64–1.73	0.836
Hospital status								
Inpatient	551	50 (9.1)	Reference	−	−	Reference	−	−
Outpatient	913	29 (3.2)	0.33	0.21–0.53	< 0.001***	0.40	0.24–0.67	< 0.001***
Chemotherapy regimen								
Breast cancer								
FEC	214	9 (4.2)	Reference	−	−	Reference	−	−
TC	208	7 (3.4)	0.81	0.30–2.14	0.664	0.90	0.34–2.41	0.831
AC/EC	216	11 (5.1)	1.21	0.50–2.93	0.671	1.49	0.61–3.64	0.377
NHL								
R-CHOP/CHOP therapy	191	12 (6.3)	1.51	0.63–3.59	0.356	0.79	0.29–2.13	0.643
NSCLC/SCLC								
Platinum-based	93	3 (3.2)	0.84	0.24–2.94	0.781	0.39	0.10–1.52	0.173
Non-platinum-based	71	1 (1.4)	0.46	0.08–2.66	0.386	0.23	0.04–1.39	0.109
Other	471	36 (7.6)	1.81	0.87–3.78	0.112	1.02	0.44–2.34	0.966
Performance status								
0	1012	39 (3.9)	Reference	−	−	Reference	−	−
1	358	26 (7.3)	1.96	1.18–3.27	0.009**	1.72	0.98–3.04	0.060
≥ 2	94	14 (14.9)	4.44	2.33–8.47	< 0.001***	3.14	1.49–6.62	0.003**
History of FN								
No	1127	49 (4.3)	Reference	−	−	Reference	−	−
Yes	337	30 (8.9)	2.16	1.35–3.45	0.001**	3.21	1.80–5.72	< 0.001***
Comorbidity (number of organ disorders)								
0	1283	63 (4.9)	Reference	−	−	Reference	−	−
1	139	9 (6.5)	1.40	0.69–2.84	0.352	1.12	0.54–2.33	0.762
≥ 2	42	7 (16.7)	4.06	1.76–9.37	0.001**	1.97	0.76–5.11	0.162
Reason for prescription								
Primary prophylaxis	743	45 (6.1)	Reference	−	−	Reference	−	−
Secondary prophylaxis	721	34 (4.7)	0.77	0.49–1.21	0.261	0.52	0.29–0.92	0.024*

^a^From the effectiveness analysis set (N = 1471), patients with missing dependent variables for this analysis (N = 7) were excluded

^*^P < 0.05; **P < 0.01; ***P < 0.001

*AC* doxorubicin hydrochloride and cyclophosphamide hydrate; *CHOP* cyclophosphamide hydrate, doxorubicin hydrochloride, vincristine sulfate, and prednisone; *CI* confidence interval, *EC* epirubicin hydrochloride and cyclophosphamide hydrate; *FEC* fluorouracil, epirubicin hydrochloride, and cyclophosphamide hydrate; *FN* febrile neutropenia, *NHL* non-Hodgkin lymphoma, *NSCLC* non-small cell lung cancer, R-CHOP rituximab, cyclophosphamide hydrate, doxorubicin hydrochloride, vincristine sulfate, and prednisone; *SCLC* small cell lung cancer; *TC* docetaxel hydrate and cyclophosphamide hydrate

## Discussion

This post-marketing surveillance study investigated the effectiveness and safety of pegfilgrastim in patients with all types of cancer and chemotherapy in a real-world setting. Our results confirmed that a single injection of 3.6 mg pegfilgrastim per cycle of chemotherapy was effective to reduce the risk of developing FN, and there were no new safety concerns in the real-world setting.

The frequency of FN was 5.6% in the overall patient group. Our study was not designed for comparison with other past research; however, the effectiveness was generally similar to studies using a 6-mg dose and different primary outcome measures [4, 12–14]. The main primary diseases in patients who received pegfilgrastim were breast cancer (51.4%), followed by NHL (25.6%), NSCLC (5.9%), and SCLC (5.2%). Other diseases were diverse (29 types of cancer) and accounted for 11.9% of patients. One purpose of this study was to describe the effectiveness in patients with cancers other than breast cancer and NHL because pegfilgrastim approval was not based on robust evidence from clinical studies. Past studies using prophylactic G-CSF showed superiority over no prophylaxis in reducing FN frequency in patients with various cancer types, such as colorectal cancer and sarcoma [10]. Although our subgroup analysis results were not conclusive owing to the small sample size, the FN frequency was generally low in patients with various cancer types.

The FN incidence rate and safety profile of pegfilgrastim in the total population might have been largely influenced by frequently used regimens. Although subgroup analysis showed reduced FN incidence across different cancer types and regimens in cycle 2 and later, the number of participants in post-hoc subgroup analysis was too small to provide sufficient power to detect a difference.

Primary prophylaxis with pegfilgrastim is now used in nearly half of perioperative chemotherapy cases for breast cancer in Japan [15]. Consistent with this real-world analysis, we observed more frequent use of pegfilgrastim for primary prophylaxis in breast cancer patients, while it was more commonly used for secondary prophylaxis in patients with other cancer types. The frequency of FN in breast cancer patients who underwent TC therapy was (5/159 patients, 3.1%) throughout the entire survey period, which was similar to the result of a phase III clinical trial in Japan (2/173 patients, 1.2%) [8].

For both primary and secondary prophylaxis subgroups, the frequency of FN was lower in cycle 2 onward compared with cycle 1, demonstrating that pegfilgrastim was effective as both primary and secondary prophylaxes. Although the population in this study was biased toward breast cancer and NHL, this tendency was also observed across patients with various cancer types and chemotherapy regimens, as shown in subgroup analysis. In most patients, neutrophil counts recovered before the next chemotherapy administration so that the cancer chemotherapy could be implemented as planned.

Administration of pegfilgrastim is not recommended within 24 h after the completion of chemotherapy because of a safety concern of exacerbating neutropenia [16–18]. In this survey, most patients (87.3%–90.5%) received pegfilgrastim between the 2nd and 4th days after chemotherapy completion, which followed the recommendation [16–18]. A small proportion of patients received pegfilgrastim on the same day as chemotherapy completion or during chemotherapy. There was no notable tendency for this deviated use from the recommendation in any specific cancer type or regimen. A prolonged period of decreased neutrophil or an increased incidence ratio of FN were concerned by the administration of pegfilgrastim on the same day of chemotherapy completion. Although the safety profile in such hematopoiesis-related disorders of these patients in our study was not largely different from that in patients treated > 24 h after the completion of chemotherapy, the number of patients was small; therefore, the safety of pegfilgrastim in such use cannot be concluded. The importance of proper administration timing (> 24 h after the completion of chemotherapy) should be further emphasized.

The risk of FN can be predicted by chemotherapy regimen, primary disease, or medical history of individual patients. Therefore, organizations such as the American Society of Clinical Oncology, European Organisation for Research and Treatment of Cancer, and National Comprehensive Cancer Network guidelines recommend assessing the risk of FN in advance of chemotherapy and using primary prophylactic G-CSF when the overall FN risk is approximately 20%, as well as considering prophylaxis in the 10%–20% range when adverse patient factors are present (e.g., age ≥ 65 years, poor PS, or prior FN) [14, 19, 20]. In addition to Japanese Society of Medical Oncology guidelines for FN whose treatment policy is similar to international guidelines [11], Japanese Society of Clinical Oncology guidelines are applied in Japan for the appropriate use of G-CSF, where a single numerical threshold is not adopted; prophylaxis is recommended based on overall clinical risk assessment [21]. Pegfilgrastim 3.6 mg once per cycle is used in Japan, and primary prophylaxis is commonly adopted in perioperative breast cancer according to regimen- and patient-level risk, which has been confirmed in real-world data analyses [15]. In this survey, we operationally defined primary prophylaxis as administration in cycle 1 and secondary prophylaxis as administration in cycle ≥ 2, acknowledging that definitions may differ in real-world clinical practice. Regarding risk factors for FN, these guidelines mention older age (≥ 65 years), advanced cancer, history of open wound surgery, history of FN, no use of G-CSF or prophylactic antimycotics, history of chemotherapy or radiotherapy, presence of neutropenia or bone marrow infiltration of tumor cells, poor PS, decreased kidney function, decreased liver function, cardiac diseases, and HIV infection [14, 19, 20]. In the current survey, subgroup analysis demonstrated that inpatients (vs. outpatients), breast cancer (vs. NSCLC/SCLC), PS 2 (vs. PS 0), and history of FN (vs. no history) had a higher risk of FN, consistent with risk factors listed in these guidelines. These observations were in concordance with the risk factors listed in the guidelines. In this survey, AEs were found in 36.4% of patients and ADRs were found in 18.5% of patients. Bone and/or back pain-related AEs were observed in 4.5% of patients and thrombocytopenia was observed in 2.9% of patients.

Bone and/or back pain-related AEs have been considered as pegfilgrastim-related. In previous clinical studies for pegylated G-CSF conducted in Japan, bone pain was found in 6.4% of treated patients with breast cancer (vs. 2.3% in the placebo group) and back pain in 19.1% of treated patients (vs. 15.0%) [8]. In patients with malignant lymphoma, bone pain was found in 0% of treated patients (vs. 9.1% in the non-pegylated G-CSF group) and back pain in 22.2% (vs. 29.1%) [9]. In the current survey, bone and/or back pain-related AEs were observed in 4.5% of all patients, including 5.3% in patients who underwent primary prophylaxis with pegfilgrastim and 3.7% in patients who underwent secondary prophylaxis with pegfilgrastim. The frequency of bone and/or back pain-related AEs in this survey was lower than those of previous clinical studies. However, the frequency of each AEs tends to be lower in post-marketing surveys because of bias in reporting; therefore, this survey cannot be compared with clinical trials. Nevertheless, bone and/or back pain-related AEs were the most commonly reported AEs after FN in this survey. In most patients with bone and/or back pain-related AEs, the onset was 2 to 7 days after pegfilgrastim administration (65.7%; median 4 days), and 14.9% of patients experienced AEs on the day of administration. It is necessary to assume that bone and/or back pain-related AEs occur and to prepare to treat them when pegfilgrastim administration is planned. All bone and/or back pain-related AEs in this survey were non-serious and well managed by medication, such as NSAIDs.

While thrombocytopenia was not designated as an AE of special interest, serious thrombocytopenia cases were observed in the past and a study using data from MID-NET® database showed an increased risk of thrombocytopenia by pegfilgrastim administration (adjusted OR 7.4 vs. no treatment; 95% CI 2.0–28.1) [22]. Therefore, we paid special attention to thrombocytopenia. Thrombocytopenia was not observed very frequently (43 patients, 2.9% in total) and most AEs were mild. Thrombocytopenia was previously observed with administration of pegfilgrastim at 3.6 mg or 7.2 mg (66.7% and 78.3%, respectively) in Japanese healthy adults; all these AEs were mild [23]. In a study conducted in the United States [24], pegfilgrastim at 30, 60, 100, or 300 µg/kg in healthy adults caused changes in platelet count from the baseline of − 11.7% (− 50%, 36%) (median (min, max) in all adults with any dose). These studies suggest a slight effect of pegfilgrastim on platelet production when administered alone and that it would be clinically not significant. Clinically significant thrombocytopenia was observed in this survey when pegfilgrastim was combined with chemotherapy regimens with strong bone marrow suppression (e.g. platinum-based chemotherapy). Thus, caution for thrombocytopenia is required depending on the chemotherapy regimen or medical history of individual patients. Nonetheless, the occurrence and frequency are as already known from previous studies and no new safety signal was observed.

## Conclusion

This real-world, post-marketing surveillance study in Japan evaluated the safety and effectiveness of pegfilgrastim 3.6 mg once every chemotherapy cycle to suppress FN in patients with various types of cancer. This survey confirmed previously demonstrated evidence from international studies regarding the effectiveness and safety of pegfilgrastim as both primary and secondary prophylaxis, also supporting its use in the Japanese real-world setting.

## Supplementary Information

Below is the link to the electronic supplementary material.

Supplementary file1 (DOCX 553 KB)

## Data Availability

Anonymized data obtained in the current survey and used for the analysis may be available upon reasonable request from a principal investigator to the corresponding author.
