# Supplementary material for: The safety and effectiveness of pegfilgrastim to reduce cancer chemotherapy-induced febrile neutropenia in real-world practice in Japan: a post-marketing surveillance study
Source: Support Care Cancer. 2025 Nov 22;33(12):1115. doi: 10.1007/s00520-025-10042-6 (PMC12640309; doi:10.1007/s00520-025-10042-6)
Supplement: Supplementary file 1 — Supplementary file1 (DOCX 553 KB) [file 520_2025_10042_MOESM1_ESM.docx]

# Title

The safety and effectiveness of pegfilgrastim to reduce cancer chemotherapy-induced febrile neutropenia in real-world practice in Japan: a post-marketing surveillance study.

# Journal

# *Supportive Care in Cancer*

# Author information

Nobuhiro Shibata^1,2^, Hiroshi Kuwazawa^3^, Tomoharu Yasukawa^3^, Manabu Iwabuchi^3^, Shigehira Saji^4^

^1^Department of Clinical Oncology, Kansai Medical University Hospital, Hirakata, Japan.

^2^Cancer Treatment Center, Kansai Medical University Hospital, Hirakata, Japan

^3^Kyowa Kirin Co., Ltd, Tokyo, Japan

^4^Department of Medical Oncology, Fukushima Medical University, Fukushima, Japan

Corresponding author:

Nobuhiro Shibata

E-mail: [shibanob.kmu@gmail.com](mailto:shibanob.kmu@gmail.com)

# Supplementary information

## Table S1 Summary of adverse events observed in ≥5 patients: Safety analysis set (N = 1479)

|  | Adverse events | | Adverse drug reactions | |
| --- | --- | --- | --- | --- |
|  | Patients  N (%) | Events N | Patients  N (%) | Events N |
| Total adverse events^a^ | 538 (36.4) | 1294 | 274 (18.5) | 530 |
| Serious adverse events | 116 (7.8) | 185 | 28 (1.9) | 34 |
| Deaths | 6 (0.4) | 7 | 1 (0.1) | 1 |
| Adverse events^b^ |  |  |  |  |
| Febrile neutropenia | 83 (5.6) |  | 11 (0.7) |  |
| Pyrexia | 64 (4.3) |  | 46 (3.1) |  |
| Back pain^c^ | 57 (3.9) |  | 53 (3.6) |  |
| Nausea | 46 (3.1) |  | 10 (0.7) |  |
| Constipation | 43 (2.9) |  | 12 (0.8) |  |
| Anemia | 39 (2.6) |  | 12 (0.8) |  |
| Arthralgia | 39 (2.6) |  | 31 (2.1) |  |
| Platelet count decreased | 35 (2.4) |  | 10 (0.7) |  |
| Neutrophil count decreased | 31 (2.1) |  | 3 (0.2) |  |
| Stomatitis | 29 (2.0) |  | 6 (0.4) |  |
| Hepatic function abnormal | 29 (2.0) |  | 22 (1.5) |  |
| Malaise | 28 (1.9) |  | 13 (0.9) |  |
| Decreased appetite | 26 (1.8) |  | 5 (0.3) |  |
| White blood cell count decreased | 26 (1.8) |  | 7 (0.5) |  |
| Myalgia | 24 (1.6) |  | 20 (1.4) |  |
| Edema | 22 (1.5) |  | 7 (0.5) |  |
| Diarrhea | 20 (1.4) |  | 5 (0.3) |  |
| Neuropathy peripheral | 19 (1.3) |  | 4 (0.3) |  |
| Taste disorder | 16 (1.1) |  | 8 (0.5) |  |
| Urticaria | 16 (1.1) |  | 12 (0.8) |  |
| Bone pain^c^ | 15 (1.0) |  | 15 (1.0) |  |
| Serious adverse events^d^ | | | | |
| Febrile neutropenia | 42 (2.8) |  | 4 (0.3) |  |
| White blood cell count decreased | 8 (0.5) |  | 0 (0.0) |  |
| Pyrexia | 6 (0.4) |  | 5 (0.3) |  |
| Platelet count decreased | 6 (0.4) |  | 2 (0.1) |  |
| Interstitial lung disease^c^ | 6 (0.4) |  | 5 (0.3) |  |
| Herpes zoster | 5 (0.3) |  | 0 (0.0) |  |
| Neutrophil count decreased | 5 (0.3) |  | 0 (0.0) |  |
| Pneumonia | 5 (0.3) |  | 0 (0.0) |  |
| Decreased appetite | 5 (0.3) |  | 0 (0.0) |  |

^a^ Multiple events in single patient were possible

^b^ Adverse events that were observed in more than 1% of patients

^c^ Adverse events of special interest

^d^ Serious adverse events that were observed in 5 or more patients

## Table S2 Thrombocytopenia (potential adverse event of special interest): Safety analysis set (N = 1479)

|  | All patients | | | Primary prophylaxis | | | Secondary prophylaxis | | |
| --- | --- | --- | --- | --- | --- | --- | --- | --- | --- |
|  | Total | Regimen with Pt | Regimen without Pt | Total | Regimen with Pt | Regimen without Pt | Total | Regimen with Pt | Regimen without Pt |
| N | 1479 | 188 | 1291 | 750 | 80 | 670 | 727 | 107 | 620 |
| Thrombocytopenia, N (%) | 43 (2.9) | 9 (4.8) | 34 (2.6) | 17 (2.3) | 1 (1.3) | 16 (2.4) | 26 (3.6) | 8 (7.5) | 18 (2.9) |
| Platelet count (lowest value), ×10^4^/µL | | | | | | | | | |
| Mean  (SD) | 6.50 (3.84) | 4.60  (3.18) | 7.00  (3.88) | 7.41  (3.68) | 4.00  (NA) | 7.62  (3.69) | 5.91  (3.89) | 4.68  (3.39) | 6.46  (4.06) |
| Median  (min, max) | 6.40  (0.1, 14.4) | 3.60  (1.5, 11.2) | 7.15  (0.1, 14.4) | 6.90  (1.8, 14.4) | 4.00  (4.0, 4.0) | 7.20  (1.8, 14.4) | 4.90  (0.1, 12.9) | 3.35  (1.5, 11.2) | 6.40  (0.1, 12.9) |
| Severity^a^ |  |  |  |  |  |  |  |  |  |
| Grade 3 (N) | 11 | 4 | 7 | 3 | 1 | 2 | 8 | 3 | 5 |
| Grade 4 (N) | 7 | 2 | 5 | 2 | 0 | 2 | 5 | 2 | 3 |
| Days after the initiation of chemotherapy until the lowest thrombocyte count | | | | | | | | | |
| Mean  (SD) | 11.9 (13.1) | 11.9  (5.2) | 11.9 (14.5) | 9.1  (2.4) | 13.0  (NA) | 8.9  (2.2) | 13.7 (16.6) | 11.8  (5.5) | 14.5 (19.7) |
| Median  (min, max) | 9.0  (4, 93) | 12.0  (7, 22) | 9.0  (4, 93) | 9.0  (4, 13) | 13.0  (13, 13) | 9.0  (4, 13) | 9.0  (7, 93) | 10.0  (7, 22) | 9.0  (7, 93) |
| Days after the initiation of pegfilgrastim until the lowest thrombocyte count | | | | | | | | | |
| Mean  (SD) | 9.0  (11.2) | 8.7  (3.6) | 9.1  (12.5) | 7.0  (2.1) | 10.0  (NA) | 6.8  (2.0) | 10.3  (14.2) | 8.5  (3.8) | 11.1  (17.0) |
| Median  (min, max) | 7.0  (3, 79) | 8.0  (5, 16) | 7.0  (3, 79) | 7.0  (3, 11) | 10.0  (10, 10) | 7.0  (3, 11) | 7.0  (5, 79) | 7.0  (5, 16) | 7.0  (5, 79) |

^a^ Severity grade definition (in ×10^4^/µL): grade 1: ≥7.5 and less than the lower limit determined by the facility; grade 2: ≥5.0 and <7.5; grade 3: ≥2.5 and <5.0; grade 4: <2.5.

*NA* not available; *Pt* platinum-based chemotherapy; *SD* standard deviation

## Table S3 Frequency of febrile neutropenia by cancer type and chemotherapy regimen: Effectiveness analysis set (N = 1471)

|  |  | Febrile neutropenia frequency: Prevalence/analyzed (%) [95% CI] | | | | | |
| --- | --- | --- | --- | --- | --- | --- | --- |
|  | Patients  N | Entire period | | Cycle 1^a^ | | |  |
| Primary prophylaxis |  |  |  |  |  |  |  |
| All cancer patients analyzed^b^ | 682 | 43/682 (6.3) | [4.6–8.4] | 36/682 (5.3) | [3.7–7.2] |  |  |
| Breast cancer | 472 | 16/472 (3.4) | [1.9–5.4] | 13/472 (2.8) | [1.5–4.7] |  |  |
| FEC | 119 | 4/119 (3.4) | [0.9–8.4] | 3/119 (2.5) | [0.5–7.2] |  |  |
| TC | 159 | 5/159 (3.1) | [1.0–7.2] | 5/159 (3.1) | [1.0–7.2] |  |  |
| AC/EC | 132 | 5/132 (3.8) | [1.2–8.6] | 3/132 (2.3) | [0.5–6.5] |  |  |
| DTX | 21 | 1/21 (4.8) | [0.1–23.8] | 1/21 (4.8) | [0.1–23.8] |  |  |
| DTX/HER | 9 | 0/9 (0.0) | [0.0–33.6] | 0/9 (0.0) | [0.0–33.6] |  |  |
| EPI | 10 | 0/10 (0.0) | [0.0–30.8] | 0/10 (0.0) | [0.0–30.8] |  |  |
| DTX/HER/PER | 5 | 0/5 (0.0) | [0.0–52.2] | 0/5 (0.0) | [0.0–52.2] |  |  |
| TCH | 8 | 0/8 (0.0) | [0.0–36.9] | 0/8 (0.0) | [0.0–36.9] |  |  |
| Other (multitherapy) | 3 | 1/3 (33.3) | [0.8–90.6] | 1/3 (33.3) | [0.8–90.6] |  |  |
| Other (monotherapy) | 6 | 0/6 (0.0) | [0.0–45.9] | 0/6 (0.0) | [0.0–45.9] |  |  |
| NHL | 94 | 17/94 (18.1) | [10.9–27.4] | 15/94 (16.0) | [9.2–25.0] |  |  |
| R-CHOP | 37 | 2/37 (5.4) | [0.7–18.2] | 1/37 (2.7) | [0.1–14.2] |  |  |
| CHOP | 11 | 2/11 (18.2) | [2.3–51.8] | 2/11 (18.2) | [2.3–51.8] |  |  |
| R-THP-COP | 7 | 2/7 (28.6) | [3.7–71.0] | 2/7 (28.6) | [3.7–71.0] |  |  |
| RIT/CPA/DOX/VCR | 2 | 0/2 (0.0) | [0.0–84.2] | 0/2 (0.0) | [0.0–84.2] |  |  |
| THP-COP | 3 | 0/3 (0.0) | [0.0–70.8] | 0/3 (0.0) | [0.0–70.8] |  |  |
| Other (multitherapy) | 34 | 11/34 (32.4) | [17.4–50.5] | 10/34 (29.4) | [15.1–47.5] |  |  |
| Other (monotherapy) | 0 | 0/0 (–) | – | 0/0 (–) | – |  |  |
| Lung cancer (NSCLC) | 28 | 2/28 (7.1) | [0.9–23.5] | 2/28 (7.1) | [0.9–23.5] |  |  |
| DTX | 5 | 0/5 (0.0) | [0.0–52.2] | 0/5 (0.0) | [0.0–52.2] |  |  |
| DTX/RAM | 5 | 0/5 (0.0) | [0.0–52.2] | 0/5 (0.0) | [0.0–52.2] |  |  |
| CBDCA/PEM/BV | 4 | 0/4 (0.0) | [0.0–60.2] | 0/4 (0.0) | [0.0–60.2] |  |  |
| CDDP/PEM | 2 | 0/2 (0.0) | [0.0–84.2] | 0/2 (0.0) | [0.0–84.2] |  |  |
| Other (multitherapy) | 11 | 2/11 (18.2) | [2.3–51.8] | 2/11 (18.2) | [2.3–51.8] |  |  |
| Other (monotherapy) | 1 | 0/1 (0.0) | [0.0–97.5] | 0/1 (0.0) | [0.0–97.5] |  |  |
| Platinum-based | 16 | 2/16 (12.5) | [1.6–38.3] | 2/16 (12.5) | [1.6–38.3] |  |  |
| Non-platinum-based | 12 | 0/12 (0.0) | [0.0–26.5] | 0/12 (0.0) | [0.0–26.5] |  |  |
| Lung cancer (SCLC) | 13 | 1/13 (7.7) | [0.2–36.0] | 1/13 (7.7) | [0.2–36.0] |  |  |
| CBDCA/VP-16 | 4 | 0/4 (0.0) | [0.0–60.2] | 0/4 (0.0) | [0.0–60.2] |  |  |
| AMR | 6 | 1/6 (16.7) | [0.4–64.1] | 1/6 (16.7) | [0.4–64.1] |  |  |
| CDDP/VP-16 | 3 | 0/3 (0.0) | [0.0–70.8] | 0/3 (0.0) | [0.0–70.8] |  |  |
| Other (multitherapy) | 0 | 0/0 (–) | – | 0/0 (–) | – |  |  |
| Other (monotherapy) | 0 | 0/0 (–) | – | 0/0 (–) | – |  |  |
| Platinum-based | 7 | 0/7 (0.0) | [0.0–41.0] | 0/7 (0.0) | [0.0–41.0] |  |  |
| Non-platinum-based | 6 | 1/6 (16.7) | [0.4–64.1] | 1/6 (16.7) | [0.4–64.1] |  |  |
| Prostate cancer | 16 | 0/16 (0.0) | [0.0–20.6] | 0/16 (0.0) | [0.0–20.6] |  |  |
| Head and neck cancer | 13 | 1/13 (7.7) | [0.2–36.0] | 1/13 (7.7) | [0.2–36.0] |  |  |
| Soft tissue tumors | 13 | 2/13 (15.4) | [1.9–45.4] | 1/13 (7.7) | [0.2–36.0] |  |  |
| Esophageal cancer | 10 | 0/10 (0.0) | [0.0–30.8] | 0/10 (0.0) | [0.0–30.8] |  |  |
| Urothelial carcinoma | 6 | 3/6 (50.0) | [11.8–88.2] | 2/6 (33.3) | [4.3–77.7] |  |  |
| Other | 17 | 1/17 (5.9) | [0.1–28.7] | 1/17 (5.9) | [0.1–28.7] |  |  |
| Secondary prophylaxis |  |  |  |  |  |  |  |
| All cancer patients analyzed^b^ | 628 | 27/628 (4.3) | [2.9–6.2] | 16/628 (2.5) | [1.5–4.1] |  |  |
| Breast cancer | 249 | 11/249 (4.4) | [2.2–7.8] | 6/249 (2.4) | [0.9–5.2] |  |  |
| FEC | 82 | 4/82 (4.9) | [1.3–12.0] | 3/82 (3.7) | [0.8–10.3] |  |  |
| TC | 39 | 1/39 (2.6) | [0.1–13.5] | 1/39 (2.6) | [0.1–13.5] |  |  |
| AC/EC | 76 | 5/76 (6.6) | [2.2–14.7] | 1/76 (1.3) | [0.0–7.1] |  |  |
| DTX | 19 | 1/19 (5.3) | [0.1–26.0] | 1/19 (5.3) | [0.1–26.0] |  |  |
| DTX/HER | 10 | 0/10 (0.0) | [0.0–30.8] | 0/10 (0.0) | [0.0–30.8] |  |  |
| EPI | 5 | 0/5 (0.0) | [0.0–52.2] | 0/5 (0.0) | [0.0–52.2] |  |  |
| DTX/HER/PER | 9 | 0/9 (0.0) | [0.0–33.6] | 0/9 (0.0) | [0.0–33.6] |  |  |
| TCH | 2 | 0/2 (0.0) | [0.0–84.2] | 0/2 (0.0) | [0.0–84.2] |  |  |
| Other (multitherapy) | 2 | 0/2 (0.0) | [0.0–84.2] | 0/2 (0.0) | [0.0–84.2] |  |  |
| Other (monotherapy) | 5 | 0/5 (0.0) | [0.0–52.2] | 0/5 (0.0) | [0.0–52.2] |  |  |
| NHL | 212 | 13/212 (6.1) | [3.3–10.3] | 7/212 (3.3) | [1.3–6.7] |  |  |
| R-CHOP | 95 | 4/95 (4.2) | [1.2–10.4] | 2/95 (2.1) | [0.3–7.4] |  |  |
| CHOP | 20 | 1/20 (5.0) | [0.1–24.9] | 0/20 (0.0) | [0.0–16.8] |  |  |
| R-THP-COP | 24 | 2/24 (8.3) | [1.0–27.0] | 1/24 (4.2) | [0.1–21.1] |  |  |
| RIT/CPA/DOX/VCR | 19 | 1/19 (5.3) | [0.1–26.0] | 1/19 (5.3) | [0.1–26.0] |  |  |
| THP-COP | 7 | 0/7 (0.0) | [0.0–41.0] | 0/7 (0.0) | [0.0–41.0] |  |  |
| Other (multitherapy) | 43 | 5/43 (11.6) | [3.9–25.1] | 3/43 (7.0) | [1.5–19.1] |  |  |
| Other (monotherapy) | 4 | 0/4 (0.0) | [0.0–60.2] | 0/4 (0.0) | [0.0–60.2] |  |  |
| Lung cancer (NSCLC) | 44 | 1/44 (2.3) | [0.1–12.0] | 1/44 (2.3) | [0.1–12.0] |  |  |
| DTX | 13 | 0/13 (0.0) | [0.0–24.7] | 0/13 (0.0) | [0.0–24.7] |  |  |
| DTX/RAM | 5 | 0/5 (0.0) | [0.0–52.2] | 0/5 (0.0) | [0.0–52.2] |  |  |
| CBDCA/PEM/BV | 4 | 0/4 (0.0) | [0.0–60.2] | 0/4 (0.0) | [0.0–60.2] |  |  |
| CDDP/PEM | 2 | 0/2 (0.0) | [0.0–84.2] | 0/2 (0.0) | [0.0–84.2] |  |  |
| Other (multitherapy) | 13 | 1/13 (7.7) | [0.2–36.0] | 1/13 (7.7) | [0.2–36.0] |  |  |
| Other (monotherapy) | 7 | 0/7 (0.0) | [0.0–41.0] | 0/7 (0.0) | [0.0–41.0] |  |  |
| Platinum-based | 20 | 1/20 (5.0) | [0.1–24.9] | 1/20 (5.0) | [0.1–24.9] |  |  |
| Non-platinum-based | 24 | 0/24 (0.0) | [0.0–14.2] | 0/24 (0.0) | [0.0–14.2] |  |  |
| Lung cancer (SCLC) | 52 | 0/52 (0.0) | [0.0–6.8] | 0/52 (0.0) | [0.0–6.8] |  |  |
| CBDCA/VP-16 | 21 | 0/21 (0.0) | [0.0–16.1] | 0/21 (0.0) | [0.0–16.1] |  |  |
| AMR | 12 | 0/12 (0.0) | [0.0–26.5] | 0/12 (0.0) | [0.0–26.5] |  |  |
| CDDP/VP-16 | 10 | 0/10 (0.0) | [0.0–30.8] | 0/10 (0.0) | [0.0–30.8] |  |  |
| Other (multitherapy) | 1 | 0/1 (0.0) | [0.0–97.5] | 0/1 (0.0) | [0.0–97.5] |  |  |
| Other (monotherapy) | 8 | 0/8 (0.0) | [0.0–36.9] | 0/8 (0.0) | [0.0–36.9] |  |  |
| Platinum-based | 34 | 0/34 (0.0) | [0.0–10.3] | 0/34 (0.0) | [0.0–10.3] |  |  |
| Non-platinum-based | 18 | 0/18 (0.0) | [0.0–18.5] | 0/18 (0.0) | [0.0–18.5] |  |  |
| Prostate cancer | 9 | 0/9 (0.0) | [0.0–33.6] | 0/9 (0.0) | [0.0–33.6] |  |  |
| Soft tissue tumors | 8 | 0/8 (0.0) | [0.0–36.9] | 0/8 (0.0) | [0.0–36.9] |  |  |
| Gastric cancer | 6 | 0/6 (0.0) | [0.0–45.9] | 0/6 (0.0) | [0.0–45.9] |  |  |
| Esophageal cancer | 6 | 0/6 (0.0) | [0.0–45.9] | 0/6 (0.0) | [0.0–45.9] |  |  |
| Ovarian cancer | 6 | 0/6 (0.0) | [0.0–45.9] | 0/6 (0.0) | [0.0–45.9] |  |  |
| Uterine cancer | 6 | 1/6 (16.7) | [0.4–64.1] | 1/6 (16.7) | [0.4–64.1] |  |  |
| Thymic cancer | 5 | 0/5 (0.0) | [0.0–52.2] | 0/5 (0.0) | [0.0–52.2] |  |  |
| Head and neck cancer | 5 | 0/5 (0.0) | [0.0–52.2] | 0/5 (0.0) | [0.0–52.2] |  |  |
| Other | 20 | 1/20 (5.0) | [0.1–24.9] | 1/20 (5.0) | [0.1–24.9] |  |  |

^a^ Cycle 1 was defined as the first cycle in which pegfilgrastim was initiated.

^b^ From the effectiveness analysis set (N = 1471), patients who received pegfilgrastim within 5 days after the completion of each chemotherapy cycle were analyzed in this summary.

*AC* doxorubicin hydrochloride and cyclophosphamide hydrate*; AMR* amrubicin hydrochloride ; *BV* bevacizumab*; CBDCA* carboplatin; *CDDP* cisplatin; *CHOP* cyclophosphamide hydrate, doxorubicin hydrochloride, vincristine sulfate, and prednisone; *CI* confidence interval *; CPA* cyclophosphamide hydrate*; DOX* doxorubicin hydrochloride *; DTX* docetaxel hydrate; *EC* epirubicin hydrocloride and cyclophosphamide hydrate; *EPI* epirubicin hydrochloride; *FEC* fluorouracil, epirubicin hydrochloride, and cyclophosphamide hydrate; *HER* trastuzumab; *NHL* non-Hodgkin lymphoma; *NSCLC* non-small cell lung cancer; *PEM* pemetrexed sodium hemipentahydrate*; PER* pertuzumab; *RAM* ramucirumab*; R-CHOP* rituximab, cyclophosphamide hydrate, doxorubicin hydrochloride, vincristine sulfate, and prednisone; *R-THP-COP* rituximab, pirarubicin, cyclophosphamide hydrate, vincristine sulfate, and prednisone; *RIT* rituximab; *SCLC* small cell lung cancer; *TC* docetaxel hydrate and cyclophosphamide hydrate; *TCH* docetaxel hydrate, carboplatin, and trastuzumab; *THP-COP* pirarubicin, cyclophosphamide hydrate, vincristine sulfate, and prednisone*; VCR*  Vincristine sulfate*; VP-16* etoposide.

## Fig. S1 Selection of patients


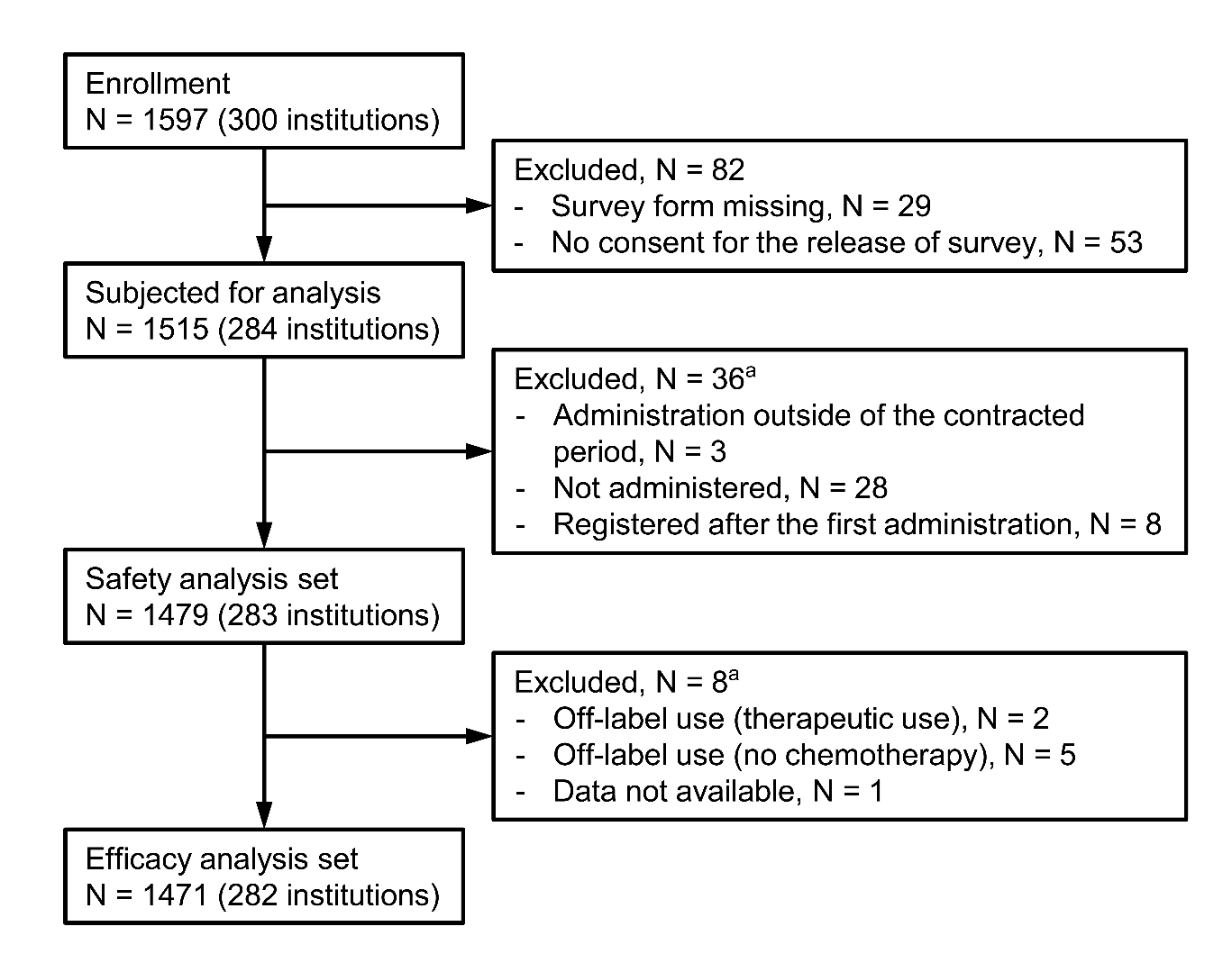


^a^ Multiple reasons in a single patient were possible

## Fig. S2 Time-course changes in neutrophil counts during treatment


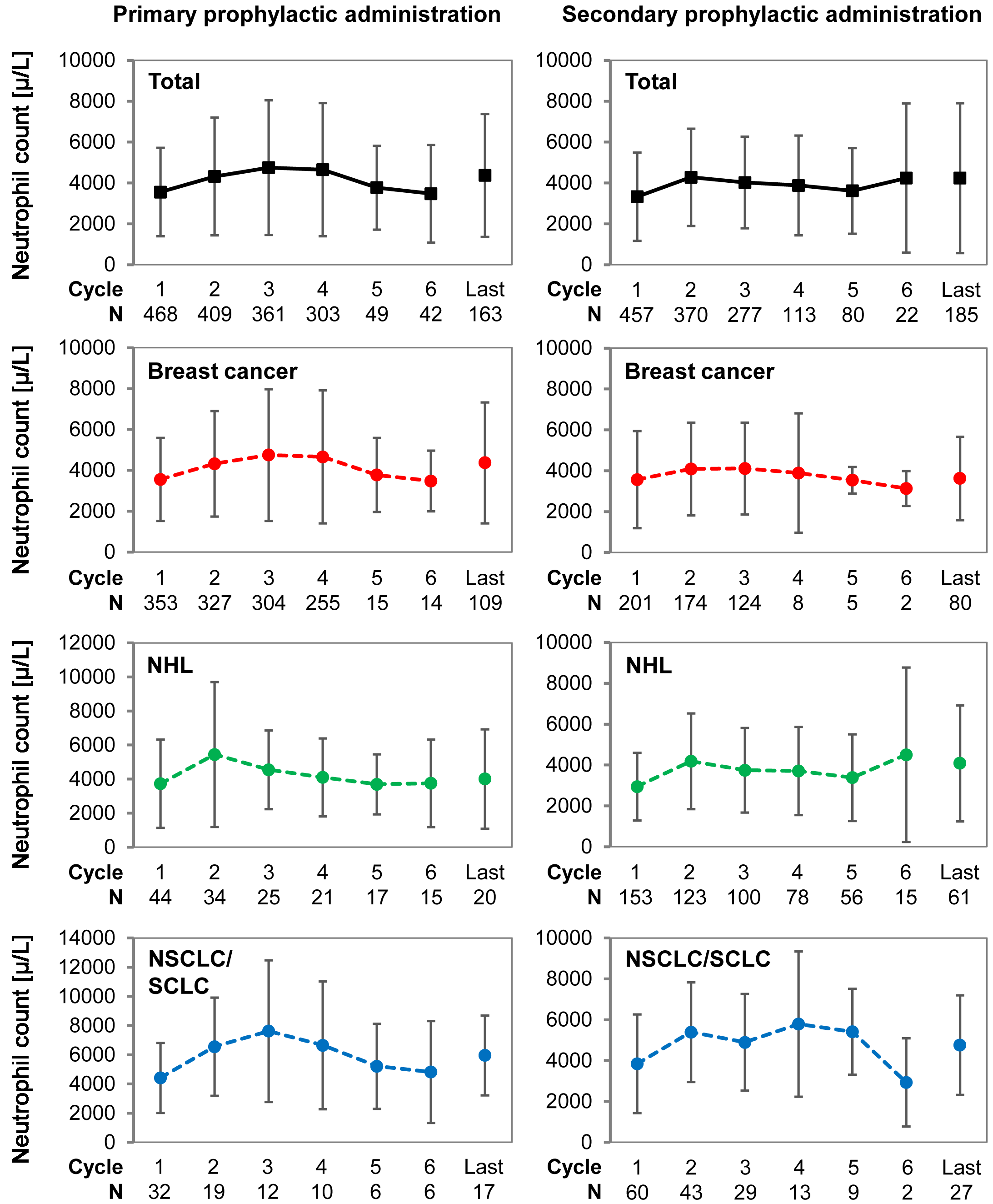


Neutrophil counts in patients with the primary prophylactic administration (left column) and the secondary prophylactic administration (right column). The count was determined before the start of chemotherapy in each cycle and at the last evaluation point.

N: number of patients analyzed, NHL: non-Hodgkin lymphoma; NSCLC: non-small cell lung cancer; SCLC: small cell lung cancer
